# Melatonin Attenuates the Urea-Induced Yields Improvement Through Remodeling Transcriptome and Rhizosphere Microbial Community Structure in Soybean

**DOI:** 10.3389/fmicb.2022.903467

**Published:** 2022-07-07

**Authors:** Renhao Xiao, Qin Han, Yu Liu, Xuehai Zhang, Qingnan Hao, Qingqing Chai, Yongfang Hao, Junbo Deng, Xia Li, Hongtao Ji

**Affiliations:** ^1^National Key Laboratory of Crop Genetic Improvement, Hubei Hongshan Laboratory, College of Plant Science and Technology, Huazhong Agricultural University, Wuhan, China; ^2^Oil Crops Research Institute, Chinese Academy of Agricultural Sciences, Wuhan, China

**Keywords:** soybean yields, foliar application, gene expression, nitrogen metabolism, microbiome

## Abstract

Foliar application of nitrogen to enhance crop productivity has been widely used. Melatonin is an effective regulator in promoting plant growth. However, the effects of melatonin and the combination of melatonin and nitrogen on soybeans yields production remain largely unknown. In this study, a field experiment was conducted to evaluate the effects and mechanisms of spraying leaves with melatonin and urea on soybeans. Foliar application of urea significantly increased soybean yields and melatonin did not affect the yields, while combination of melatonin and urea significantly reduced the yields compared to the application of urea alone. A leaf transcriptional profile was then carried out to reveal the underlying mechanism and found that foliar spraying of urea specifically induced the expression of genes related to amino acid transport and nitrogen metabolism. However, foliar application of melatonin significantly changed the transcriptional pattern established by urea application and increased the expression of genes related to abiotic stress signaling pathways. The effects of melatonin and urea treatment on soil microbiome were also investigated. Neither melatonin nor urea application altered the soil microbial alpha diversity, but melatonin application changed rhizosphere microbial community structure, whereas the combination of melatonin and urea did not. Melatonin or urea application altered the abundance of certain taxa. The number of taxa changed by melatonin treatment was higher than urea treatment. Collectively, our results provide new and valuable insights into the effects of foliar application of melatonin to urea and further show that melatonin exerts strong antagonistic effects on urea-induced soybean yields, gene expression and certain soil microorganisms.

## Introduction

Nitrogen fertilizers are used to increase global crop yields in the last half century (Lassaletta et al., 2014; Garnett et al., 2015), whereas excessive nitrogen fertilization does not further promote crop productivity but lead to environmental pollution. In China, the overuse of nitrogen fertilizer has become one of the major issues in recent years. Thus, it is necessary to explore new strategies for improving nitrogen utilization efficiency without increasing nitrogen fertilizers application. Melatonin (*N-*acetyl-5-methoxytryptamine) is a crucial plant growth regulator and regulates plant growth and development, response to various abiotic stresses, nitrogen uptake and metabolism (Arnao and Hernández-Ruiz, 2014, 2019). However, whether melatonin could promote plant nitrogen utilization efficiency and crop yields remain large unknown.

Melatonin (*N-*acetyl-5-methoxytryptamine) was first discovered in the bovine pineal gland of animals, and it was also shown to be present in higher plants and involved in plant growth and development regulation (Lerner et al., 1958; Hattori et al., 1995). Plant melatonin not only regulates many physiological processes but also acts as a protective agent against multiple stresses. It was reported that melatonin regulated seed germination, root growth, flowering, photosynthesis and leaf senescence (Zhang et al., 2012; Byeon and Back, 2014; Wei et al., 2015). Melatonin could also protect plants against multiple biotic and abiotic stresses (Arnao and Hernández-Ruiz, 2015; Tiwari et al., 2021). Treatment with exogenous melatonin significantly improves the resistance of plants to salt stress (Zheng et al., 2017), heat stress (Zhang J. et al., 2017), chilling (Balabusta et al., 2016), and drought stress (Cao et al., 2020) through remodeling a series of physiological and transcriptomic profiles. More important, melatonin has been shown to promote plant nutrient uptake and metabolism under various stresses condition. Melatonin application promotes nitrogen metabolism in alfalfa exposed to drought stress (Antoniou et al., 2017). Zhang R. et al. (2017) also found that melatonin improved the tolerance of cucumber seedlings to high nitrate levels by enhancing the activities of nitrogen metabolism-related enzymes. Melatonin also significantly decreases the melon leaf NH_4_^+^ content by improving the activities of enzymes involved in nitrogen metabolism under chilling stress (Gao et al., 2016). Recently, Qiao et al. (2019) reported that melatonin promotes winter wheat growth and yield by increasing the activities of nitrogen uptake and metabolism-related enzymes under nitrogen-deficient conditions. These studies emphasized the role of melatonin in promoting plant nutrient uptake and metabolism under stresses condition, whereas the effects of melatonin on crop plant nitrogen uptake and metabolism under normal nitrogen condition remain elusive.

Soil nitrogen fertilization is commonly used to enhance crop productivity, but soil nitrogen fertilization not only affects soil properties but also directly affects the abundance of members of the soil microbial community, including bacteria, fungi and other microorganisms (Chu et al., 2007; Wang et al., 2017). The impact of soil nitrogen fertilization on soil bacterial diversity has been studied in maize (Rodríguez-Blanco et al., 2015), rice (Zhang et al., 2019), wheat (Chen et al., 2019), and soybean (Liu et al., 2019). In addition, the foliar application of urea is another method of nitrogen fertilization to enhance crop productivity (Wagan et al., 2017). Foliar urea application has some advantages compared to soil application especially for the legume crops; crops can absorb foliar-applied urea even in dry weather conditions, and the absorption of urea can reduce the loss of nitrogen to soil (Gooding and Davies, 1992). For legume crops, foliar application could reduce the inhibition role of nitrogen to root nodule development and symbiotic nitrogen fixation (Lin et al., 2018; Nishida et al., 2018). Thus, foliar application of nitrogen fertilizer has become more popular, and the physiological effects of foliar urea application on crop plants have been explored extensively in recent years (Hassanein et al., 2019; Cassim et al., 2020; Lyu et al., 2021). In wheat, foliar urea application altered storage protein distribution of grain by promoting the expression of the majority of storage protein-encoding genes (Rossmann et al., 2019), but the global transcriptional profile of soybean after foliar urea application has rarely been reported.

The changes in leaf transcription patterns that occur after foliar urea treatment also induce changes in the rhizosphere microbiome. Using culture-independent methods, González-Arenzana et al. (2017) found that foliar application of urea significantly alters the microbial diversity and population structure of the must, but did not affect grape microbiota. Foliar application of urea to the aerial parts of wheat increased the abundance of bacteria and decreased the abundance of some fungal genera on the roots of plants grown in uncontaminated soil and soil contaminated with the noxious fungus *Fusarium* spp. (Vran, 1972). In soybean, the effects of foliar nitrogen application on the root and soil microbiomes have rarely been explored, and the role of foliar melatonin application on the rhizosphere microbiome community of soybeans is also unreported.

Soybean, an important legume worldwide, is a major source of oil and protein for people and livestock (Gaonkar and Rosentrater, 2019). Effective agroecosystem practices to enhance nitrogen use efficiency, increase the yields and explore the underlying mechanism remain challenges. In this study, we tried to figure out whether melatonin could further promote soybean yields under urea condition and investigate the underlying mechanism. Through a field experiment by spraying soybean with melatonin and/or urea in two places, we confirmed that additional melatonin application resulted a significant decrease of soybean yields, altered the leaf gene expression pattern, and changed certain soil microorganisms compared with urea application alone.

## Materials and Methods

### Plant Materials and Treatments

Experiments were conducted at Jingmen (latitude 30°52′29″, longitude 112°11′31″) and Yangluo (latitude 30°43′8″, longitude 114°30′27″) experimental base of the Chinese Academy of Agricultural Sciences, Hubei, China. The soil physicochemical properties of both fields are clay with medium soil fertility and flat terrain. In Jingmen base, the soil pH is 5.53, organic matters are 21.39 g⋅kg^–1^, soil available potassium is 183 mg⋅kg^–1^, soil available phosphorus is 44.5 mg⋅kg^–1^ and soil total nitrogen is 1.06 g⋅kg^–1^. In Yangluo station, the soil pH is 8.11, organic matters are 15.94 g⋅kg^–1^, soil available potassium is 136.29 mg⋅kg^–1^, soil available phosphorus is 11.68 mg⋅kg^–1^ and alkali-hydrolyzable nitrogen is 87.41 mg⋅kg^–1^. The two soybean cultivars (ZD63, Zhongdou 63; and WD28, Wandou 28) were sowed on 9th June, 2020 with a plant-spacing of 10 cm and row spacing of 50 cm, respectively. To ensure the germination rate, more seeds were sowed in the field and the plants were thinned to a final stand of 200,000 plants ha^–1^ within 3 weeks to meet space requirement. When soybean grown at R1 (beginning of the flowering stage, fifth unrolled trifoliate leaf) stages on 22th July, and R3 [Beginning pod—pods are 3/16 inch (5 mm) at one of the four uppermost nodes] on 24th August, the experimental plants were divided into four groups as follows: (i) the control group, which was treated with water; (ii) the urea group, which was treated with 3 kg ha^–1^ urea by leaf spraying (on the top and bottom sides of all leaves until water dripped). (iii) the melatonin group, which was treated with 100 μmol⋅L^–1^ melatonin by leaf spraying. (iv) the urea + melatonin group, which was treated with 3 kg⋅ha^–1^ urea plus 100 μmol⋅L^–1^ melatonin by leaf spraying. The urea was bought from Sinopharm Chemical Reagent Co., Ltd., company (CAS: 57-13-6), the melatonin was diluted into distilled water (without surfactant) to make 100 μmol⋅L^–1^ melatonin solution. The plants were treated for a total of two times (at R1 and R3 growth stage). Each treatment was repeated in three plots of 7.5 m^2^ each (3 × 2.5 m^2^). Soil samples from root rhizosphere were then collected at 12th August and 2nd September, the trifoliate leaves were also harvested simultaneously. The soil and leaves samples were stored at −80°C for microbiome analysis and transcriptomic analysis.

### Transcriptomic Analysis RNA Extraction

Total RNA was extracted from 100 mg trifoliate leaves (samples were collected on 2nd September from Yangluo base) using TRIzol^®^ Reagent (Plant RNA Purification Reagent for plant tissue) according the manufacturer’s instructions (Invitrogen). The genomic DNA was removed using DNase I (TaKara). The RNA quality was determined by 2100 Bioanalyser (Agilent) and quantified using the ND-2000 (NanoDrop Technologies). Only high-quality RNA sample (OD260/280 = 1.8∼2.2, OD260/230 ≥ 2.0, RIN ≥ 6.5, 28S:18S ≥ 1.0, > 1 μg) was used to construct sequencing library.

### Library Preparation, and Illumina Hiseq xten/Nova seq 6000 Sequencing

RNA-seq transcriptome libraries were prepared following TruSeqTM RNA sample preparation Kit from Illumina (San Diego, CA, United States) using 1 μg of total RNA. Firstly, messenger RNA was isolated according to polyA selection method by oligo(dT) beads and then fragmented by fragmentation buffer. Secondly, double-stranded cDNA was synthesized using a SuperScript double-stranded cDNA synthesis kit (Invitrogen, CA, United States) with random hexamer primers (Illumina). Then the synthesized cDNA was subjected to end-repair, phosphorylation and “A” base addition according to Illumina’s library construction protocol. Libraries were size selected for cDNA target fragments of 300 bp on 2% Low Range Ultra Agarose followed by PCR amplified using Phusion DNA polymerase (NEB) for 15 PCR cycles. After quantified by TBS380, paired-end RNA-seq sequencing library was sequenced with the Illumina HiSeq xten/NovaSeq 6000 sequencer (2 × 150 bp read length). Statistical methods were used to calculate the base distribution and quality fluctuation of each cycle of all sequenced reads, which could intuitively reflect the library construction quality and measurement of sequenced samples from a macro-perspective.

### Read Mapping

The raw paired end reads were trimmed and quality controlled by SeqPrep^1^ and Sickle^2^ with default parameters. During this step, clean data (clean reads) were obtained by removing reads containing adapters or poly-N sequences as well as low-quality reads. Additionally, Q20 and Q30 values and the GC content of the clean data were calculated. Then clean reads were separately aligned to reference genome with orientation mode using HISAT2^3^ software (Kim et al., 2015). The mapped reads of each sample were assembled by StringTie^4^ in a reference-based approach (Pertea et al., 2015).

### Differential Expression Analysis and Functional Enrichment

To identify differential expression genes between two different samples, the expression level of each transcript was calculated according to the transcripts per million reads (TPM) method. RNA-Seq by Expectation Maximization (RSEM)^5^ was used to quantify gene abundances (Dewey and Li, 2011). DESeq2 (Love et al., 2014) provides statistical routines for detecting differential expression in digital gene expression data, which uses a model based on the negative binomial distribution. The resulting *P* values were adjusted using the Benjamini and Hochberg approach for controlling the false-discovery rate (FDR). Genes with P adjust < 0.05 found by DESeq2 and log2 (fold change) ≥ 1 were assigned as differentially expressed. Functional-enrichment analysis including GO was performed to identify which DEGs were significantly enriched in GO terms at Bonferroni-corrected *P*-value ≤ 0.05 compared with the whole-transcriptome background. GO functional enrichment was carried out by Goatools^6^. We then explored the molecular functions of 234,966 genes in each cluster using GO annotations, 174,190 genes for cellular component and 639,419 genes for biological process GO annotations.

### Quantitative RT-PCR

To validate the RNA-seq results, the total RNA used for RNA-seq was also reverse-transcribed into cDNA with Hifair^®^ II 1st Strand cDNA Synthesis SuperMix (YEASEN, CA, United States). Different expression genes were selected and verified by RT-qPCR using SYBR Green PCR Master Mix (YEASEN, CA, United States) and the Bio-Rad CFX384 PCR detection system. The relative mRNA level of each target gene was evaluated against soybean elongation factor 1-beta (*GmELF1b*) (Glyma.02G276600). The primers were shown in Supplementary Table 4. Three technical replicates were performed and the relative levels of transcript abundance were calculated using the 2^–Δ^ ^Δ^ *^CT^* method.

### 16S rRNA Gene Sample Preparation, Sequencing and Analysis

In total, 48 rhizosphere soil (24 samples were collected on 12th August and 24 samples were collected on 2nd September) and 4 bulk soil samples (mixed samples collected on 12th August and 2nd September) from Yangluo base were used for sequencing according to standard protocols at Personal Biotechnology Co., Ltd., (Shanghai, China). Total genomic DNA samples were extracted using the OMEGA Soil DNA Kit (D5625-01) (Omega Bio-Tek, Norcross, GA, United States) following the manufacturer’s instructions. The quantity and quality of extracted DNAs were measured using a NanoDrop ND-1000 spectrophotometer (Thermo Fisher Scientific, Waltham, MA, United States) and agarose gel electrophoresis, respectively. The V3-V4 target region of 16S rRNA gene fragments were amplification and sequenced on an Illumina Novaseq PE250 sequencing platform (Illumina, San Diego, United States).

Sequence data analyses were mainly performed using QIIME2 2019.4 (Bolyen et al., 2018) and R packages (v3.2.0). Alpha diversity indices, such as Chao1, Observed species, and Shannon were calculated using the non-singleton amplicon sequence variants (ASV) table in QIIME2, and visualized as box plots. Beta-diversity (between-sample diversity) was estimated by Bray-Curtis distance and the differentiation of microbiota structure among groups was assessed by PERMANOVA (Permutational multivariate analysis of variance) in QIIME2. LEfSe (Linear discriminant analysis effect size) was performed to detect differentially abundant taxa across groups using the default parameters (Segata et al., 2011). Quantitative real-time PCR amplifications (qPCR) were used to determine the abundances of total bacteria (the primers Eub338F/Eub518R) in the bulk soil and rhizosphere according to Nadkarni et al. (2002) and Fierer et al. (2005). Standard curves were generated using 10-fold serial dilutions of *Escherichia coli* DNA sample.

### Soybean Yields Evaluation

Five square meters plot containing 100 plants were used for yield evaluation. The seeds from three plots were weighted individually, and the variance analysis was performed by using GraphPad prism 8.0 software. The data are reported as the mean and standard error (SE) values of three biological replicates. Ordinary one-way analysis of variance (ANOVA) and Tukey’s multiple comparisons tests were used to determine the significance of the differences among samples.

## Results

### Melatonin Reduces the Effect of Urea on Increasing Soybean Yields

**FIGURE 1:**
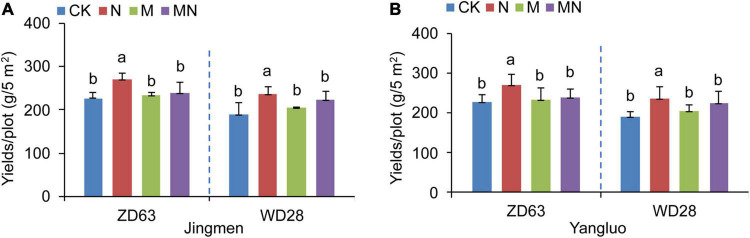
The yields of two soybean cultivars under different treatments. **(A,B)** The yields of Zhongdou 63 (ZD63) and Wandou 28 (WD28) after foliar application of melatonin and/or urea at Jingmen **(A)** and Yangluo **(B)** field station. CK, Control; N, foliar application of urea alone; M, foliar application of melatonin alone; MN, foliar application of both melatonin and urea. Data indicates means ± SE with One-way ANOVA analysis. Different letters indicate significant differences.

Nitrogen is an important macronutrient required for plant growth and crop productivity. Melatonin is reported to be one of the most widely used plant growth-promoting reagent. To investigate whether melatonin application could further promote soybean yields under urea fertilization conditions, we sprayed soybean leaves with both melatonin (M treatment, 100 μmol⋅L^–1^) and urea (N treatment, 3 kg⋅ha^–1^). Our yields evaluation results showed that foliar application of urea (N treatment, 3 kg⋅ha^–1^) to two cultivars of Chinese soybean (Zhongdou 63, ZD63; Wandou 28, WD28) significantly increased their yields when compared with the yields of seedlings exposed to control conditions in two fields (Figures 1A,B). However, spraying exogenous melatonin alone could not further improve soybean yields. On the contrary, foliar application of both melatonin and urea (MN treatment) resulted in a lower production than the urea treatment (Figures 1A,B). In the two experimental fields, the yields of the two cultivars showed the same trend in response to melatonin and urea fertilizer treatment (Figures 1A,B). These results indicated that spraying leaves with melatonin significantly reduced the effect of urea on increasing soybean yields.

### Identification of Differentially Expressed Genes in Response to Melatonin and Urea Treatments

To further study the molecular mechanism underlying the effects of melatonin and nitrogen on soybean yields, we performed RNA-seq analysis. Eleven ZD63 leaf samples treated with urea (N), melatonin (M) and MN (melatonin and urea together) were collected for transcriptome analysis. A total of 491,611,474 raw reads were generated from these samples, and the average number of raw reads for each sample was 44,691,952. An average of 97.89% of the raw reads had a quality score of Q30 (an error probability for base calling of less than 0.1%). After filtering and trimming the raw reads, 486,975,816 high-quality reads were used for further analysis and an average of 93.71% of the raw reads had a quality score of Q30 (Supplementary Table 1). The clean data from each sample were mapped to the *Glycine max* Wm82.a4 reference genome sequence with TopHat2 HISAT2 software. The proportion of total mapped reads ranged from 90.92 to 96.22% (Supplementary Table 2). Based on Pearson’s correlation analysis, there were significant differences between the control and different treatments. The controls and MN samples are high correlated between biological replicates (R^2^ > 0.89). The correlation of two urea samples is 0.85. The correlations among three melatonin samples are 0.77, 0.80 and 0.95, respectively (Supplementary Figure 1).

**FIGURE 2:**
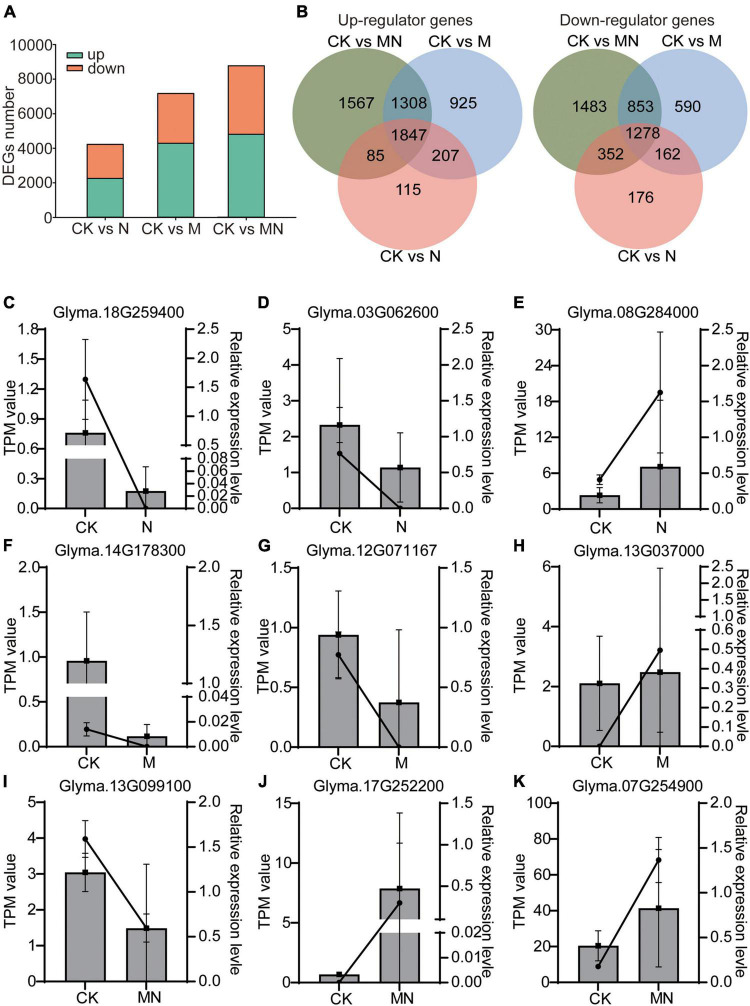
Differentially expressed genes in responsive to different treatments. **(A)** Total number of upregulated (green) and downregulated (orange) DEGs identified for N, M, and MN treatments compared with the control. **(B)** Venn diagram depicting the number of upregulated and downregulated DEGs. **(C–K)** The mRNA abundance of nine DEGs using qRT-PCR assay. Lines represent the results of the transcriptome data (TPM value); column charts represent the results of qRT-PCR. Data shown means ± SD (*n* = 3 replicates/group).

We then identified the differentially expressed genes (DEGs) from different foliar spray treatments. The number of DEGs was calculated compared with the control group (the threshold for all the comparisons was DEGs ≥ 2-fold change; P adjust < 0.05). Compared with the control, the urea treatment produced 4,222 DEGs in total, with 2,254 DEGs being upregulated and 1,968 DEGs being downregulated. A total of 7,170 DEGs were identified in response to melatonin treatment, including 4,287 upregulated and 2,883 downregulated DEGs. MN treatment resulted in a maximum amount of 8,773 DEGs, including 4,807 upregulated and 3,966 downregulated DEGs (Figure 2A and Supplementary Table 3). Venn diagram analysis of these DEGs among the different treatment groups revealed 1,847 upregulated and 1,278 downregulated DEGs as shared DEGs among the N, M and MN treatment groups (Figure 2B). However, when the transcriptome profiles induced by the three different treatments were compared, each treatment was found to induce unique differences in gene expression; 115 upregulated DEGs and 176 downregulated DEGs were uniquely associated with urea treatment, 925 upregulated DEGs and 590 downregulated DEGs were only observed after melatonin treatment, and 1,567 upregulated DEGs and 1,483 downregulated DEGs were specifically observed in response to MN treatment. We then performed qRT-PCR to validate the RNA-seq results. As shown in Figures 2C–K, the nine selected genes displayed the similar expression pattern with RNA-seq data. These results indicated that the RNA-seq data is convincing. All these DEGs might be responsive to respective treatments and subsequently affect plant growth and yield production.

### Foliar Application of Melatonin and Urea Influence Multiple Biological Processes

**FIGURE 3:**
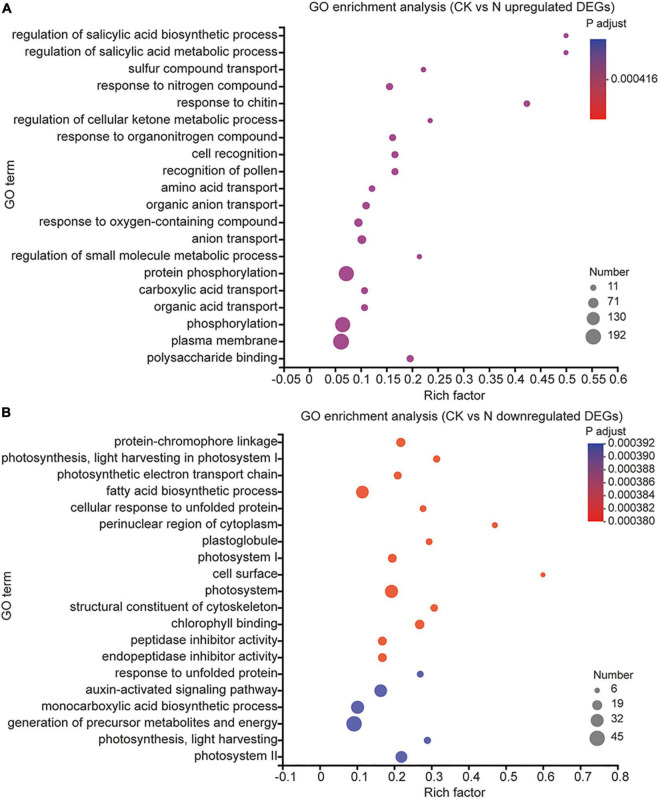
GO enrichment analysis of DEGs under urea treatments. **(A)** The GO terms were enriched from upregulated DEGs. **(B)** The GO terms were enriched from downregulated DEGs.

To understand the possible biological processes in which these DEGs are involved, we then performed gene ontology (GO) term^7^ enrichment analysis of all 11,005 DEGs (Supplementary Table 5). The top 20 enrichments in urea treatment groups are shown in Figure 3. We found that the upregulated DEGs were involved in “regulation of salicylic acid biosynthetic and metabolic process, response to chitin, response to nitrogen compound, response to organonitrogen compound, cell recognition, recognition of pollen,” and “organic acid transport,” and the downregulated DEGs were involved in “protein-chromophore linkage, photosynthesis,” and “light harvesting in photosystem I”; The above-listed GO items were also found in melatonin and MN treatment (Supplementary Figures 2, 3), which indicated that melatonin and/or urea treatment regulated some common biological processes. In the individual urea treatment group, the GO term “amino acid transport” was specifically upregulated, which suggested that urea might be catalyzed by urease and reutilized through amino acid transport biological processes (Figure 3A). We also found that urea treatment downregulated genes associated with “photosynthetic electron transport chain, fatty acid biosynthetic process and auxin-activated signaling pathway.” The decrease in the carbon fixation/metabolism and auxin signaling pathway might be explained by the reduction of plant growth and promotion the seed filling (Figure 3B). However, the DEGs associated melatonin treatment involved in different biological processes compared with those associated with urea treatment. Upregulated DEGs under melatonin treatment involved in the biological processes of “response to hydrogen peroxide, second-messenger-mediated signaling, calcium-mediated signaling, response to wounding,” and “response to heat” (Supplementary Figure 2). All these processes represent the reported roles of melatonin in regulating plant growth and responses to various abiotic stresses. When the melatonin and urea treatments were combined, we found that the addition of the melatonin significantly altered the urea-induced changes in gene expression and biological processes (Supplementary Figure 3); in addition to the above-listed common biological processes which existed in all three treatments, the “response to wounding” biological processes was found in melatonin treatment and MN treatment, but not in the urea treatment, indicating that the addition of melatonin induces genes expression related to abiotic stress pathway. We also found that under MN treatment, the specifically upregulated GO terms were “cell death, xyloglucan metabolic process, cell wall macromolecule metabolic process,” and “protein ubiquitination” (Supplementary Figure 3A), which further indicated that the addition of melatonin might trigger a stress response and subsequently affect crop growth and yield production.

### Melatonin Alters Nitrogen Uptake or Metabolic Biological Processes

**FIGURE 4:**
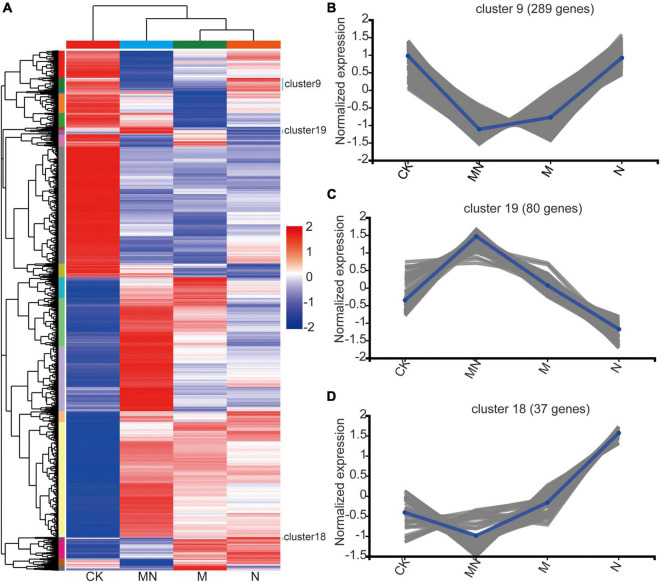
The expression pattern of DEGs among different treatments. **(A)** All DEGs were clustered and shown in a heat map. **(B–D)** Normalized expression pattern of DEGs from cluster 9, 19 and 18. CK, Control; N, foliar application of urea alone; M, foliar application of melatonin alone; MN, foliar application of both melatonin and urea.

Urea notably increased the expression of genes related to amino acid transport, whereas melatonin induced the expression of genes related to hydrogen peroxide, second messenger-mediated signaling, and abiotic stress processes. These results led us to hypothesize that melatonin might play an antagonistic role in the context of urea application. To further understand the different effects of the melatonin and urea treatments, we performed hierarchical clustering using silhouette plots to assess the expression patterns of all 11,005 DEGs associated with all the treatments (Figure 4A). The heat maps were then created on Majorbio Cloud Platform^8^. Top twenty clusters were formed, and the clusters in the control treatment groups were significantly different from those in the melatonin, urea and MN treatment groups (Figure 4A and Supplementary Table 6). MN treatment has more differences than melatonin or urea treatment, which demonstrated that additional melatonin treatment significantly altered the transcriptional patterns induced by urea treatment. For example, we found that the DEGs in three clusters (Cluster 9, Cluster 19 and Cluster 18) had similar expression patterns in response to the melatonin and MN treatments compared with urea treatment. A total of 289 DEGs in Cluster 9 were downregulated in response to the melatonin and MN treatments, and these DEGs were not affected by the urea treatment (Figure 4B). In Cluster 19, 80 DEGs that were downregulated in response to urea treatment were highly expressed in response to the melatonin and MN treatments (Figure 4C). Cluster 18 contained 37 DEGs that were upregulated in response to the urea treatment, while these DEGs were downregulated in response to the melatonin and MN treatments (Figure 4D). These results indicated that the melatonin and urea treatments induced different DEGs and affect different physiological pathways.

**FIGURE 5:**
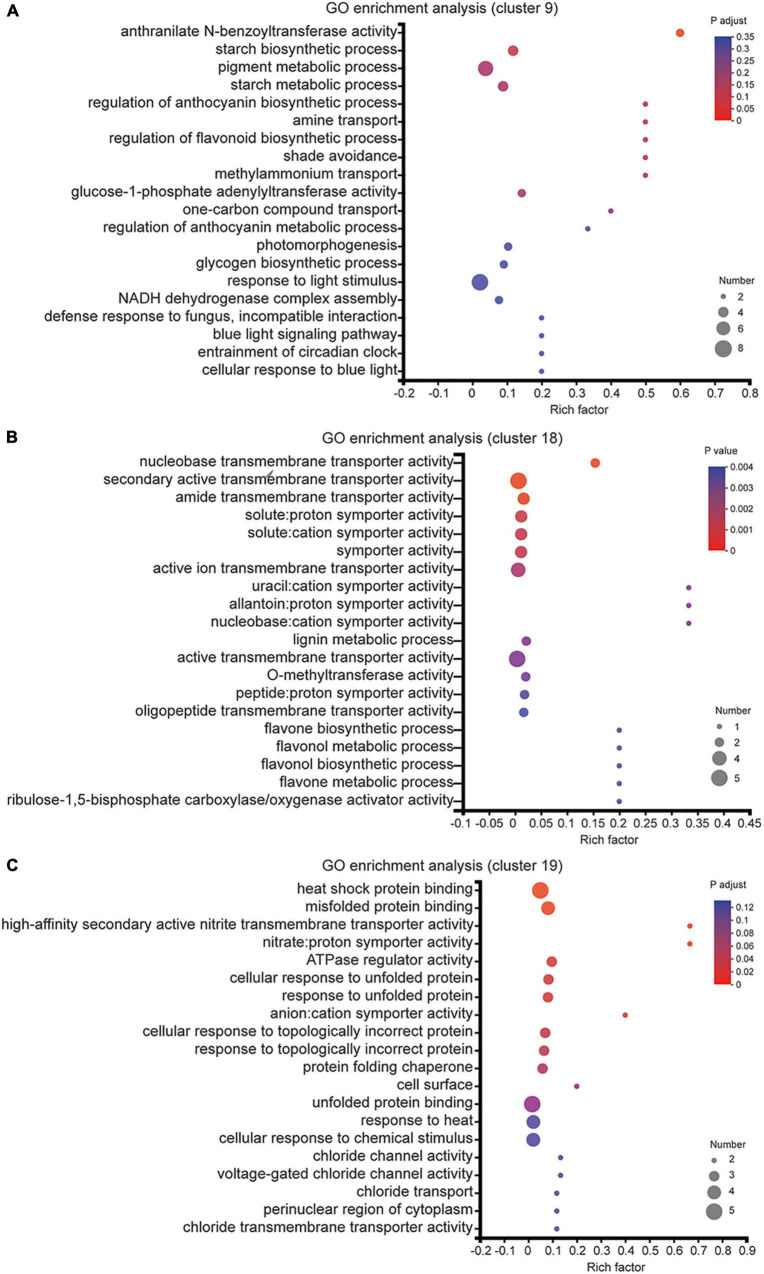
GO enrichment analysis of DEGs from cluster 9, cluster 18 and cluster 19. **(A–C)** The enriched GO terms of cluster 9 **(A)**, cluster 18 **(B)** and cluster 19 **(C)** DEGs.

We also utilized the GO enrichment tool to examine the functions of these DEGs in Clusters 9, 18 and 19. In Cluster 9, all genes displayed lower expression level under melatonin and MN treatment than urea treatment (Figure 4B), and these genes were involved in “starch biosynthetic process (GO:0019252),” “amine transport (GO:0015837),” “methylammonium transport (GO:0015843)” and “glycogen biosynthetic process (GO:0005978)” (Figure 5A), indicating that repression of these biological processes by melatonin and MN treatment might reduce the growth of soybean and yields production; In Cluster 18, all genes showed highest expression level in response to urea treatment (Figure 4D). The genes were involved in “lignin metabolic process,” “flavone biosynthetic process (GO:0051554)” and several transporters activity-related processes (Figure 5B), indicating that these genes might be beneficial for soybean development in response to urea treatment. In Cluster 19, all genes showed highest expression level in response to both melatonin and MN treatment (Figure 4C). We found that “heat shock protein binding (GO:0031072),” “misfolded protein binding (GO:0051787)” and unfolded protein binding related biological processes were highly enriched in Cluster 19 (Figure 5C). These highly expressed genes in response to melatonin and MN treatment might repress soybean growth and reduce yields.

### Melatonin Affects the Microbial Community Diversity in Soybean Rhizosphere

**FIGURE 6:**
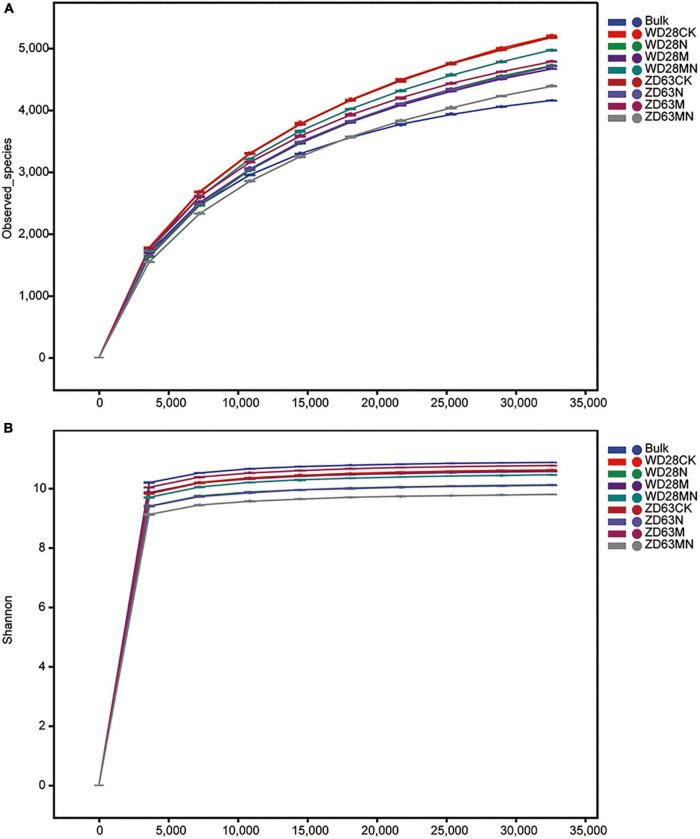
Rarefaction curves of rhizosphere samples with different treatments. **(A,B)** The rhizosphere samples were collected from Yangluo **(A)** and Jinmen **(B)** field station, where two cultivars WD28 and ZD63 were planted. WD28CK or ZD63CK indicates normal growth WD28 or ZD63 without treatment; WD28M or ZD63M indicates foliar application of melatonin to WD28 or ZD63; WD28N or ZD63N indicates foliar application of urea to WD28 or ZD63; WD28MN or ZD63MN indicates foliar application of both melatonin and urea to WD28 or ZD63.

Rhizosphere microbe play an important role in plant growth and yield. Firstly, we quantified bacterial numbers in rhizosphere samples using a general bacterial primer pair targeted at the *16S rRNA* gene. We found four treatments (ZD28N, ZD63N, ZD63M and ZD63NM) significantly reduced bacterial target numbers in rhizosphere samples compared to the bulk soil, but there was no significant difference among other treatments (Supplementary Figure 4). This result indicated that urea and melatonin treatment did not affect the total number of bacteria in rhizosphere soil. To further evaluate whether the effects of urea and melatonin on the yield of were related to rhizosphere microbe, we detected the microbial community diversity and structure of rhizosphere samples collected from Yangluo base by 16S rRNA sequencing. In total, 5,273,449 (average of 101,412) high-quality 16S rRNA gene (V3-V4) reads were obtained from 52 samples, and 3,136,811 (average of 60,323) ASVs were obtained. Rarefaction curves based on species richness (observed species and Shannon index) showed clear asymptotes and plateau levels for the different samples (Figures 6A,B); these results indicated that the sampling depth was adequate. Across all the samples, we detected approximately 27 phyla, 78 classes, 156 orders, 246 families and 391 genera. The top ten phyla were Acidobacteria, Proteobacteria, Acidobacteria, Chloroflexi, Cyanobacteria, Firmicutes, Bacteroidetes, Gemmatimonadetes, Patescibacteria and Rokubacteria.

**FIGURE 7:**
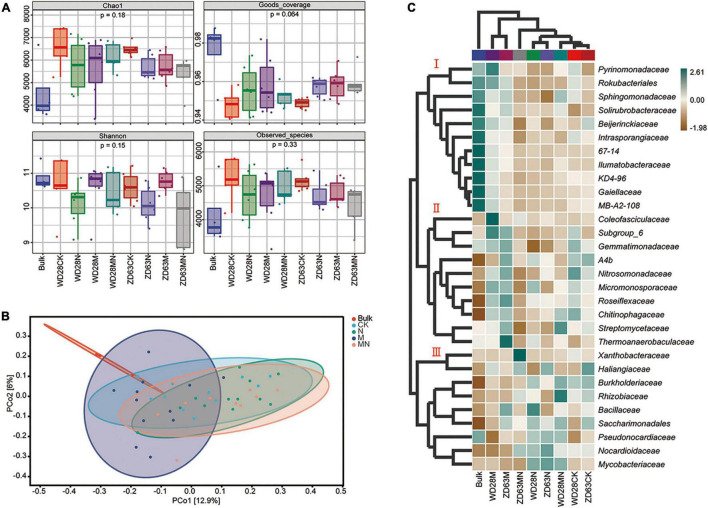
Effects of urea and melatonin on microbial diversity and composition in soybean rhizosphere. **(A)** The alpha diversity (Chao1, Goods_coverage, Shannon, Observed_species) analysis of samples. **(B)** PCoA analysis based on Bray-Curtis distances of the different treatment of rhizosphere sample; Clustering significance by treatment was determined by Adonis [Pr (> F) = 0.001]. **(C)** Composition and clustering of bacterial microorganisms from different rhizosphere samples on the Family level.

Combining two sampling times, we found that the alpha diversity indexes including Chao1, Goods_coverage, Shannon and Observed_species were not significantly different among the different treatment groups (Figure 7A), which indicated that the foliar urea and melatonin treatments did not affect rhizosphere microbial alpha diversity. We then conducted beta-diversity (PCoA) analysis to detect differences in structure among samples. The PCoA showed that the rhizosphere samples from the bulk soil and treatment groups were clearly separated (Figure 7B), indicating that planting soybean plants can affect the microbial community structure of soil. However, the rhizosphere samples from the control and urea treatment groups were not clearly separated (Figure 7B), and PERMANOVA based on the Bray-Curtis distances showed that the differences were not significant (Table 1); these results suggested that foliar urea treatments did also not affect the rhizosphere microbial community structure. For the melatonin treated samples, the rhizosphere samples were significantly separated from the control, urea treatment and urea plus melatonin treatment. The PERMANOVA based on the Bray-Curtis distances showed that the differences between melatonin and other three treatments were significant (Table 1); these results suggested that foliar urea treatments did not affect the rhizosphere microbial community structure, but melatonin did. For the urea and melatonin treatment, we found that the microbial community structure was similar to that treated with urea alone, but significantly different from that treated with melatonin (Table 1). It suggested that the effect of melatonin on rhizosphere microbial structure began to weaken when urea was added.

**TABLE 1:** PERMANOVA analysis of the rhizosphere microbial community in different group based on Bray–Curtis.

Group 1	Group 2	Sample size	Permutations	Pseudo F	*P*-value	*Q*-value
All	–	49	999	1.932	0.001	–
Bulk	CK	15	999	2.830	0.003	0.005
Bulk	N	16	999	3.684	0.001	0.005
Bulk	M	16	999	2.038	0.003	0.005
Bulk	NM	14	999	3.332	0.002	0.005
CK	N	23	999	1.165	0.128	0.160
CK	M	23	999	1.462	0.016	0.023
CK	NM	21	999	1.096	0.215	0.239
N	M	24	999	2.279	0.001	0.005
N	NM	22	999	1.029	0.329	0.329
M	NM	22	999	1.803	0.002	0.005

### The Effect of Melatonin on Rhizosphere Microbial Species Was Greater Than That of Urea

**FIGURE 8:**
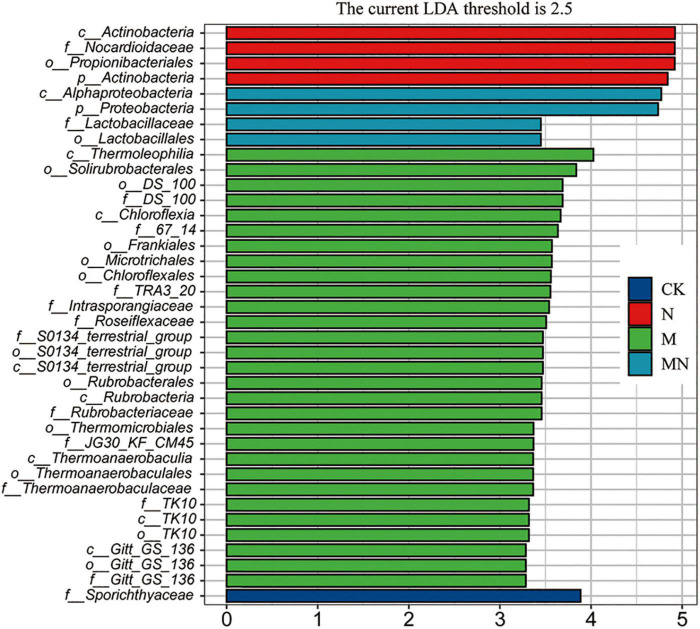
Effects of nitrogen and melatonin on microbe species in soybean rhizosphere. Linear discriminant analysis (LDA) coupled with the effect size measurements identifies the significant abundance of data in different treatment. Only taxa with LDA values greater than 2.5 (*P* < 0.05) were shown.

To further determine the trends of the changes in microbial abundance among the various treatment groups, we conducted a cluster analysis at the family level. The result showed that samples exposed to the same treatment obviously clustered together, and the top 30 families could be divided into three main clusters, which hosted distinct bacterial assemblages (Figure 7C). Among them, the abundance of cluster 1 was higher in the bulk soil, the abundance of taxa in cluster 2 was higher in melatonin treatment groups, and the abundance of taxa in cluster 3 was the highest in the urea and melatonin plus urea treatment groups. The taxa enriched in the control samples include cluster 2 and cluster 3. Finally, through linear discriminant analysis (LEfSe, LDA log score threshold > 2.5, abundance > 0.001 and *P* < 0.05), we found that the *f__Nocardioidaceae* abundance increased after urea treatment. Nevertheless, the abundance of a large number of taxa was altered after melatonin treatment. For example, the relative abundances of *f*__*DS_100*, *f__67_14*, *f*__*TRA3_20*, *f__Intrasporangiacea*e, *f*__*Roseiflexaceae*, *f*__S0134_ terrestrial_group, *f__Rubrobacteriaceae*, *f*__*JG30_KF_CM45*, *f*__*Thermoanaerobaculaceae*, *f*__*TK10* and *f*__*Gitt_GS_136* were altered. When urea and melatonin were treated together, the changed microorganisms became less (Figure 8). These results indicated that the effect of urea on rhizosphere microorganisms was less than that of melatonin, urea treatment only changed the abundance of a few microorganisms which led to little effect on microbial community structure; while melatonin treatment increased the abundance of microorganisms, which led to significant changes in microbial community structure. The abundance of microorganisms decreased when urea plus melatonin treatment was applied, but the yield did not increase (Figure 1). It suggested that the response of underground microbial community to foliar urea and melatonin treatment was independent of yield.

## Discussion

Nitrogen is an essential nutrient for plant growth and crop yields. Melatonin has been reported to promote nitrogen uptake and assimilation and increase plant growth (Qiao et al., 2019), but few studies have explored the possible effects of melatonin and nitrogen treatment on soybean with detailed transcriptomic and microbiome analyses. In this study, a combination of transcriptomic and microbiome analyses was performed to highlight the effect of exogenous melatonin and urea on field growth soybean. Field experiments revealed that foliar application of both melatonin and urea decreased soybean yields compared with application of urea alone (Figure 1). Our transcriptome analysis revealed that urea treatment induced genes expression related to amino acid transport and nitrogen compound response processes, while melatonin application may exert its opposite effects mainly through regulation the genes expression related to signal transduction and stress response pathways (Figure 3 and Supplementary Figures 2, 3). The combined application of melatonin and urea significantly altered the gene expression pattern compared with the urea treatment (Figure 4). The microbiome analysis also demonstrated that melatonin not urea treatments affected the rhizosphere microbial community structure, and the combined application of melatonin and urea showed similar microbial community structure to that treated with urea alone. Both melatonin and urea treatment changed the abundance of several microorganisms (Figures 7, 8). This study highlighted the possible antagonistic effects of melatonin application on the transcriptomic and soil microorganisms compared with urea treatment in soybean.

A transcriptional approach was first used to explain the antagonistic effects between melatonin and urea treatment. Foliar application of melatonin or urea could induce some similar and different changes at the transcriptional level in soybean (Figure 3). Urea or melatonin treatment affected several common biological processes, such as responsive to salicylic acid biosynthetic and metabolic process, chitin, nitrogen compound and organonitrogen compound, indicating overlapping functions of these two chemical compounds. However, a significant difference was also observed. Our Venn diagram sorting, hierarchical clustering, and GO enrichment analysis revealed some specific DEGs were induced upon different treatments. It is noteworthy that urea treatment affected amino acid transport biological process specifically, which was different from melatonin treatment. We found some DEGs under melatonin and MN treatments displayed similar expression pattern compared with urea treatment (Figures 4C,D), for example, DEGs in two clusters (Cluster 9 and Cluster 18) decreased expression in response to the melatonin and MN treatments compared with urea treatment (Figure 4). We then took a close look at the gene expression under melatonin and urea treatment and found that the expression levels of the *GmNRT2.5* (*Glyma.08G284000* and *Glyma.18G141900*) genes which involved in nitrate transport were upregulated in the urea treated samples (Figure 2E). While under melatonin treatment, the genes involved in abiotic stresses were mainly induced. For example, the expression of genes related to wounding responses *GmJAR6* (*Glyma.06G243500*), heat responses *GmHSF18* (*Glyma.09G143200* and *Glyma.16G196200*), and *GmHSP20* (*Glyma.20G015900, Glyma.07G043600* and *Glyma.16G012000*) were highly induced in RNA-seq data (Figure 4A and Supplementary Table 3). When melatonin and urea were both applied to soybean, obviously different transcription patterns were observed. Additional melatonin application induced the expression of genes related to salt stress response (*Glyma.10G253000*) and cell death (*GmTIR2*; *Glyma.06G268700, Glyma.08G301200* and *Glyma.09G075500*). These transcriptional differences between melatonin and urea treatment might partially explain the reduced soybean yields by additional application melatonin to urea treatment, implying that melatonin was not beneficial for soybean growth and yields production when foliar application it at grain filling stage. In addition, it was clearly demonstrated that urea is converted to amino acids in leaves after foliar application, and root nitrogen uptake is enhanced by the application of urea to leaves of apple trees (Dong et al., 2015), we speculated that foliar melatonin application might also affect the leaf nitrogen metabolism and root nitrogen uptake. Based on the transcriptomic results and soybean yields production under melatonin treatment, we might carefully choose an appropriate growth stage of soybean for melatonin foliar application, and the melatonin concentration should also be considered.

Rhizosphere or root microorganisms play an important role in the soil nitrogen cycle and are beneficial to the absorption of nitrogen by plants (Hacquard et al., 2015). In turn, nitrogen in the soil also affects the microbial community structure in the soil (Zeng et al., 2016), but the response of the microbial structure in the rhizosphere or root to different forms or concentrations of nitrogen [nitrate (NO_3_^–^-N, NH_4_^+^-N) and organic nitrogen] varies in soil and water environments (Gallart et al., 2018; Zhao et al., 2020). Generally, nitrogen levels in the soil mainly increased the abundance of *Proteobacteria* and *Bacteroidetes* but decreased the abundance of *Acidobacteria* and *Planctomycetes* (Kavamura et al., 2018; Liu et al., 2019; Zhou et al., 2021). In wheat, high levels of inorganic nitrogen in soil were found to have a significantly negative effect on bacterial richness and diversity, leading to a less stable bacterial community structure over time (Kavamura et al., 2018). In leguminous plants, application of excessive levels of nitrogen compounds to soil strongly inhibits nodule formation and changes the rhizosphere microbial community structure. Foliar nitrogen application can enhance crop yields; but it can alleviate the effect of nitrogen on soil properties. In our study, we found that the abundance of only a few taxa (such as *Nocardioidaceae*) were altered after nitrogen treatment through linear discriminant analysis (Figure 7). At the community level, we found that foliar application of urea reduced the abundance and diversity of rhizosphere microorganisms, but the difference was not significant (Figure 7A). In addition, foliar application of urea had no effect on the structure of rhizosphere microorganisms (Figure 8). These results suggested that compared to soil nitrogen application, aboveground nitrogen application has little effect on underground rhizosphere microorganisms. Altogether, our findings addressed the interesting question about whether the benefits associated with foliar urea application can be attributed to meeting the nutritional demand of the crop plant for nitrogen without restructuring the soil microbial community assemblage.

However, the effect of melatonin treatment on rhizosphere microorganisms was obviously different from that of urea treatment. Melatonin treatment did not change the soil microbial alpha diversity, but it did alter rhizosphere microbial community structure and the abundance of a larger number of taxa than urea treatment (Figure 8). Melatonin inhibited the abundance of *Nocardioidaceae* but increased the abundance of *f*__*DS_100*, *f__67_14*, *f*__TRA3_20, *f*__*Intrasporangiaceae*, *f*__Roseiflexaceae, *f*__*S0134*_*terrestrial*_*group*, *f*__*Rubrobacteriaceae*, *f*__*JG30_ KF_CM45*, *f*__*Thermoanaerobaculaceae*, *f*__*TK10* and *f*__*Gitt_GS_136*, resulting in significant changes in the community structure of rhizosphere microorganisms. Interestingly, melatonin did not change the structure of rhizosphere microbial community when urea was applied at the same time, but it could affect the promotion role of urea on yield. Melatonin had been shown to reprogram gut microbiota communities in animals and humans (Ghareghani et al., 2018; Ren et al., 2018). In mice, melatonin can reverse high-fat diet-induced lipid accumulation and gut microbiota alterations (Yin et al., 2018). The effect of melatonin on rhizosphere microbial community structure has not been reported in soybean, and the mechanism is unknown. We speculated that melatonin may affect the structure of rhizosphere microorganisms by affecting plant physiological metabolism.

In conclusion, we proved that melatonin could not further promote soybean yields under urea treatment, and revealed the possible mechanism by transcriptomic and microbiome analyses. Foliar application of melatonin induced the expression of a set of genes in soybean leaves and changed rhizosphere microbial community structure which different from urea treatment, which might influence the soybean yields. Thus, to develop melatonin as a plant growth regulator that can be practically used in crop production, optimized agricultural practices including the crop performance, application method and plant growth stage still required further exploration.

## Data Availability Statement

The data presented in this study are deposited in the National Genomics Data Center repository, accession number: PRJCA009060 (https://ngdc.cncb.ac.cn/bioproject/).

## Author Contributions

HJ conceived and designed the project. RX, QH, YL, XZ, QGH, QC, YH, and JD performed the experiments. RX and QH analyzed the data. RX, QH, and HJ wrote the manuscript. All authors read and approved the final manuscript.

## Conflict of Interest

The authors declare that the research was conducted in the absence of any commercial or financial relationships that could be construed as a potential conflict of interest.

## Publisher’s Note

All claims expressed in this article are solely those of the authors and do not necessarily represent those of their affiliated organizations, or those of the publisher, the editors and the reviewers. Any product that may be evaluated in this article, or claim that may be made by its manufacturer, is not guaranteed or endorsed by the publisher.

## Footnotes

1
https://github.com/jstjohn/SeqPrep

2
https://github.com/najoshi/sickle

3
http://ccb.jhu.edu/software/hisat2/index.shtml

4
https://ccb.jhu.edu/software/stringtie/index.shtml?t=example

5
http://deweylab.biostat.wisc.edu/rsem/

6
https://github.com/tanghaibao/Goatools

7
http://geneontology.org/

8
www.majorbio.com

## Supplementary Material

Supplementary Figure 1 Correlation analysis between samples. CK, Control; N, foliar application of urea alone; M, foliar application of melatonin alone; MN, foliar application of both melatonin and urea. The colors represent the correlation coefficient (red color indicates higher correlation and blue color indicates lower correlation).

Supplementary Figure 2 GO enrichment analysis of DEGs under melatonin treatments. **(A)** The GO terms were enriched from upregulated DEGs. **(B)** The GO terms were enriched from downregulated DEGs.

Supplementary Figure 3 GO enrichment analysis of DEGs under urea and melatonin treatments. **(A)** The GO terms were enriched from upregulated DEGs. **(B)** The GO terms were enriched from downregulated DEGs.

Supplementary Figure 4 The quantification of total bacteria in rhizosphere samples. The quantification of total bacteria in rhizosphere samples. qPCR results of the different treatments were analyzed with Newman-Keuls Multiple Comparison Test tests (*n* = 3, * indicates *P* < 0.05). WD28CK or ZD63CK indicates normal growth WD28 or ZD63 without treatment; WD28M or ZD63M indicates foliar application of melatonin to WD28 or ZD63; WD28N or ZD63N indicates foliar application of urea to WD28 or ZD63; WD28MN or ZD63MN indicates foliar application of both melatonin and urea to WD28 or ZD63.

Supplementary Table 1 Raw data statistics.

Supplementary Table 2 Quality control data statistics.

Supplementary Table 3 Differentially expressed genes.

Supplementary Table 4 qRT-PCR primers.

Supplementary Table 5 GO enrichment analysis of CK compared with each differern treatment.

Supplementary Table 6 GO enrichment analysis of different clusters.
