# Supplementary figures and images for: Melatonin Attenuates the Urea-Induced Yields Improvement Through Remodeling Transcriptome and Rhizosphere Microbial Community Structure in Soybean
Source: Front Microbiol. 2022 Jul 7;13:903467. doi: 10.3389/fmicb.2022.903467 (PMC9301482; doi:10.3389/fmicb.2022.903467)

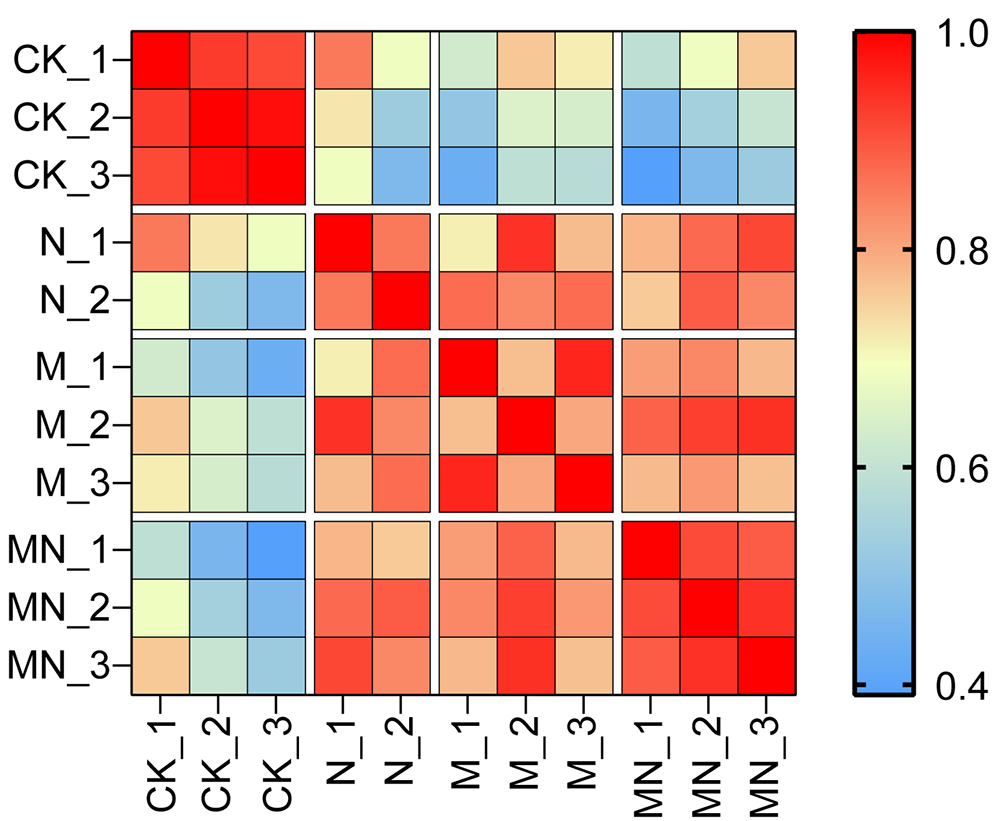

Supplement: Supplementary Figure 1 — Correlation analysis between samples. CK, Control; N, foliar application of urea alone; M, foliar application of melatonin alone; MN, foliar application of both melatonin and urea. The colors represent the correlation coefficient (red color indicates higher correlation and blue color indicates lower correlation). [file Image_1.tif]

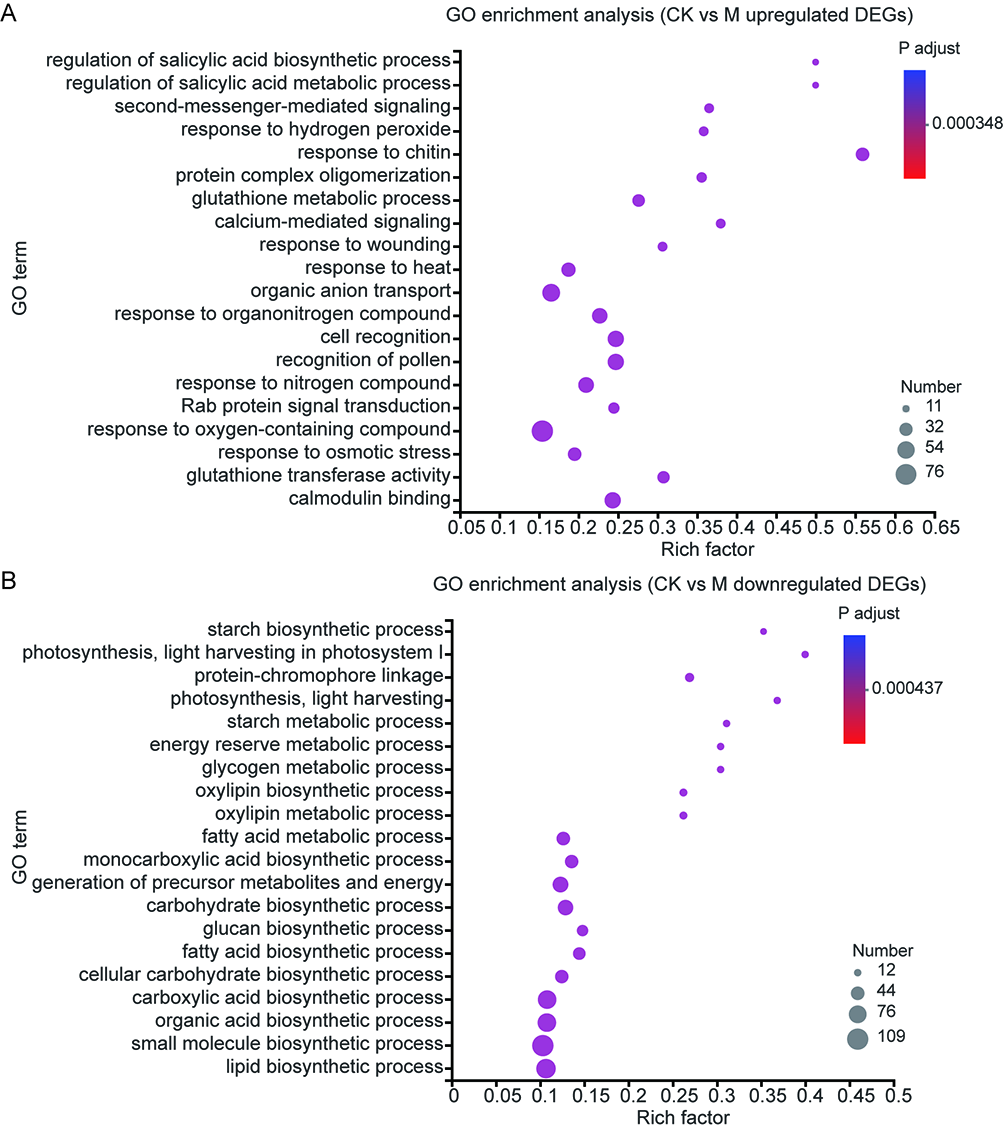

Supplement: Supplementary Figure 2 — GO enrichment analysis of DEGs under melatonin treatments. (A) The GO terms were enriched from upregulated DEGs. (B) The GO terms were enriched from downregulated DEGs. [file Image_2.tif]

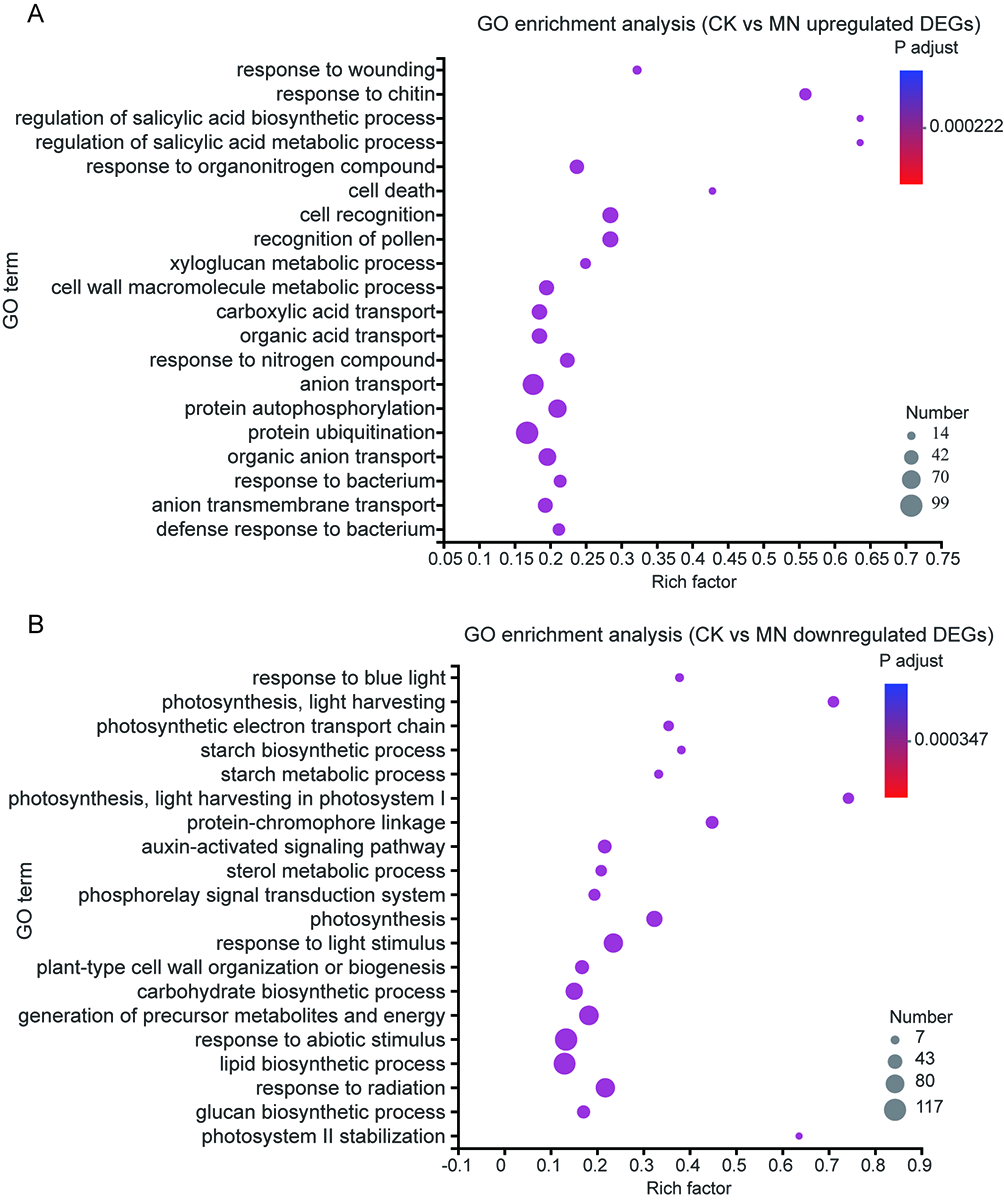

Supplement: Supplementary Figure 3 — GO enrichment analysis of DEGs under urea and melatonin treatments. (A) The GO terms were enriched from upregulated DEGs. (B) The GO terms were enriched from downregulated DEGs. [file Image_3.tif]

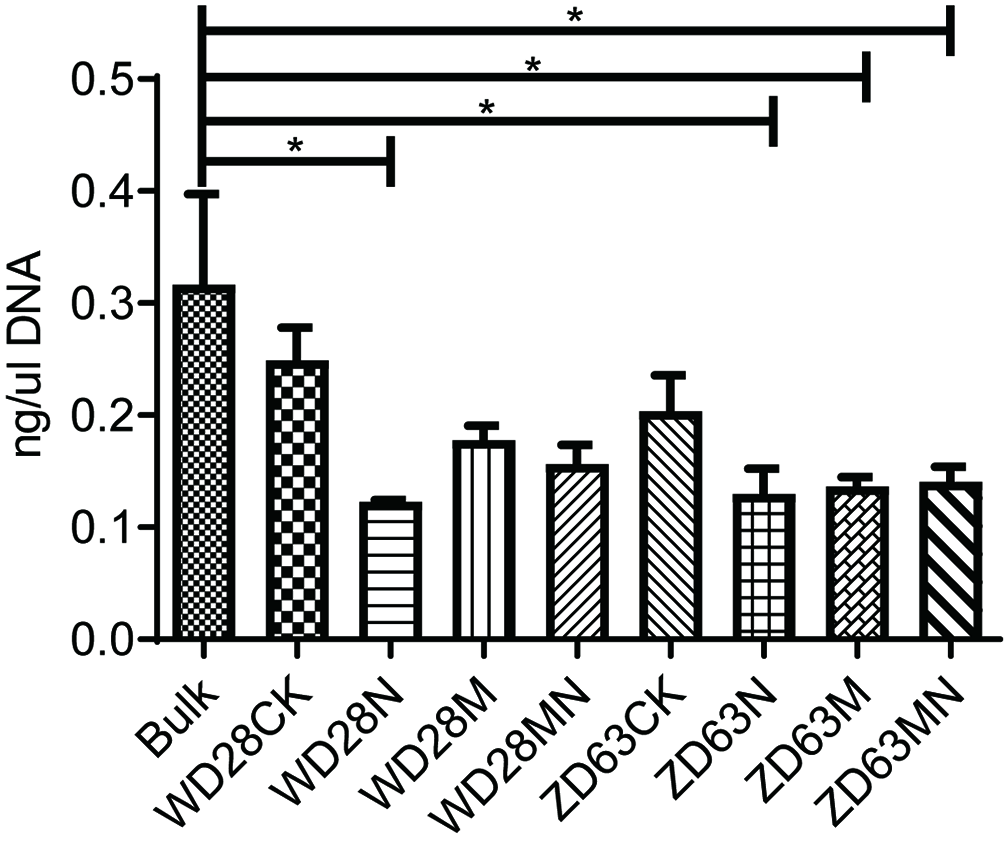

Supplement: Supplementary Figure 4 — The quantification of total bacteria in rhizosphere samples. The quantification of total bacteria in rhizosphere samples. qPCR results of the different treatments were analyzed with Newman-Keuls Multiple Comparison Test tests (n = 3, * indicates P < 0.05). WD28CK or ZD63CK indicates normal growth WD28 or ZD63 without treatment; WD28M or ZD63M indicates foliar application of melatonin to WD28 or ZD63; WD28N or ZD63N indicates foliar application of urea to WD28 or ZD63; WD28MN or ZD63MN indicates foliar application of both melatonin and urea to WD28 or ZD63. [file Image_4.tif]
